# Reply to Fry, L.E.; MacLaren, R.E. Comment on “Di Giosaffatte et al. A Novel Hypothesis on Choroideremia-Manifesting Female Carriers: Could *CHM* In-Frame Variants Exert a Dominant Negative Effect? A Case Report. *Genes* 2022, *13*, 1268”

**DOI:** 10.3390/genes14122161

**Published:** 2023-11-29

**Authors:** Niccolò Di Giosaffatte, Paola Grammatico, Irene Bottillo

**Affiliations:** Division of Medical Genetics, Department of Experimental Medicine, San Camillo-Forlanini Hospital, Sapienza University, Circ. Gianicolense, 87, 00152 Rome, Italy; niccolo.digiosaffatte@uniroma1.it (N.D.G.); paola.grammatico@uniroma1.it (P.G.)

We express our gratitude to Dr. Fry and Prof. McLaren [1] for their feedback on our recent paper [2]. Indeed, immediately after the diagnosis of choroideremia in the family, our first thought was to attribute the clinical picture of the female carriers (i.e., cases I:2 and II:2) to an unfavorable skewing of X inactivation (XCI). However, we found this possibility, at least from our perspective, to be unusual. In fact, although X inactivation skewing is common in females, usually there is no significant concordance between mothers and daughters regarding which of the two X is predominantly inactivated [3]. Therefore, by chance, both the females in our family should have inactivated the X carrying the wild-type allele in the majority of the retinal cells. This could be possible, but there is not a proper way to demonstrate it since the XCI assay performed on the other tissue could not be reliable for the retinal XCI ratio estimation. Hence, we hypothesized other possible inheritable modifier factors that could have caused a worsening of the phenotype in both women. The evidence surrounding the potential partial escape from XCI of *CHM* caught our attention because, at the single cell level, that could imply a possible perturbing effect of the in-frame variant on the activity of the wild-type REP1 allele.

Dr. Fry and Prof. McLaren’s observations are based on three main points: (i) the evidence for a potential partial escape from XCI of *CHM* is circumstantial and subject to possible confounding factors; (ii) if *CHM* is expressed biallelically, the number of female carriers without fundus anomaly would be greater because of the presence of a functioning REP1 isoform translated by the wild-type allele in all retinal cells; (iii) if the mechanism of the CHM:c.1349 + 1G > A variant is dominant negative, a more severe phenotype would be expected in the affected male, consistent with the more severe phenotype in female carriers.

Regarding point (i), our colleagues state that when inferring a biallelic *CHM* expression through massive parallel sequencing, as in references [4,5], the possible non-specific alignment on *CHM* of reads coming from homologous coding region sequences of the *CHML* gene should be properly excluded. Even though the studies performed in references [4,5] used both quantitative (comparison of the number of aligned reads) and qualitative (maternal/paternal allele-specific expression through informative SNPs) approaches, hence somewhat limiting this opportunity, they were not meant to specifically infer the *CHM* status. Thus, we agree that studies performed exclusively for *CHM* are warranted.

Regarding point (ii), the authors’ observation assumes that the expression of *CHM* from the inactivated X (Xi) is equal to that of the active X (Xa), representing a complete escape from XCI. However, the current (limited) evidence regarding the variable XCI escape of *CHM* suggests that the *CHM* allele expression on Xi is likely lower than its counterpart on Xa [6]. Therefore, in cells where the X chromosome carrying the wild-type allele is inactivated, the level of functional REP1 would still be significantly lower compared to cells in which the X chromosome carrying the mutated allele is inactivated. This could still result in a deficit in Rab prenylation, nonetheless. In this perspective, regardless of the specific type of pathogenic variant, we can infer that the damage to the retinal tissue would still be evident even in the presence of partial *CHM* escape. Moreover, the percentage of cells that inactivate the wild-type allele would continue to play a crucial role in the severity of the clinical presentation. The partial escape mechanism would, therefore, not serve as a substitute for the effect of unbalanced XCI in determining the degree of severity but rather as an additional phenotype modifier that could worsen the disease course only in the presence of in-frame variants acting with a negative dominant effect. This condition would likely represent a small minority of the cases.

Finally, the authors suggest that the putative dominant negative effect of the c.1349 + 1G > A variant should have also caused a more severe phenotype in the male patient. The dominant negative effect of a variant relies on the condition that the mutated protein, in some manner, diminishes the activity of its wild-type counterpart. Regarding choroideremia, if *CHM* is biallelically expressed, this could occur through competition for enzyme and/or substrate binding. While the presence of partial *CHM* escape could make it possible in the mutated females, in the hemizygous male carrying only the mutated allele, there is no wild-type counterpart to compete with; the final effect would exclusively be a complete abolition of the REP1 function. Functionally speaking, the final effect of the c.1349 + 1G > A variant in males is the same as every null variant. This point is explained in Figure 7 in [2].

Regarding the extent of retinal involvement in the male of our family, the authors also observe that the presence of the preserved central foveal region in the right eye is consistent with a milder phenotype, suggesting that the variant may have a less pronounced pathogenic effect compared to a null variant. However, the preservation of a substantial foveal area is limited to one eye, not both. In our view, if the variant had a milder effect, it would be more likely to produce a milder phenotype in both eyes. Moreover, if the variants were hypomorphic, it might be expected that, even in the presence of XCI skewing in the two females, they would present a milder phenotype compared to those with skewing and null variants. Lastly, if the c.1349 + 1G > A had a less severe effect than the null variants, this would mean that, regardless of the model considered (classic vs. alternative), some levels of Rab prenylation by RGGT are maintained. Thus, the c.1349 + 1G > A would encode for a REP1 protein that would be able to maintain the bond with Rab. Our in silico predictions contradict this hypothesis, but we acknowledge that they remain to be demonstrated in vitro.

In conclusion, we are once again very grateful to Dr. Lewis E. Fry and Prof. Robert E. MacLaren for the opportunity to further discuss our results. Since our hypothesis currently relies on a single family, we propose that both retrospective and prospective cohort studies should be conducted to strengthen the relevance of this potential new genotype–phenotype correlation. The goal would be to confirm, in female carriers matched for the same age and onset of fundus alterations, the association between the predicted in silico effect of specific *CHM* in-frame variants (those that impair the REP1/Rab interaction without altering REP1/RGGT binding) and the presence of a more severe phenotype (geographical or male-pattern, as described in [7]). This may require a robust international multicenter effort, as missense/in-frame splicing variants represent only a minority of the total *CHM* cases and female carriers. This could be important to evaluate potential limitations in the current therapeutic strategies, such as gene augmentation therapy, which may not lead to an optimal surrogate level of functioning REP1 in the presence of a variant exerting a dominant negative effect.
